# Does Needle Design Affect the Regenerative Potential of Bone Marrow Aspirate? An In Vitro Study

**DOI:** 10.3390/life11080748

**Published:** 2021-07-26

**Authors:** Nadia Feddahi, Monika Herten, Tjark Tassemeier, Heike Rekasi, Alexander Hackel, Marcel Haversath, Marcus Jäger

**Affiliations:** 1 Department of Orthopedic and Trauma Surgery, University Hospital Essen, University of Duisburg-Essen, Hufelandstrasse 55, D-45147 Essen, Germany; Heike.Rekasi@uk-essen.de (H.R.); Marcel.Haversath@tissueflow.com (M.H.); 2 Clinic of Trauma, Hand and Reconstructive Surgery, University Hospital Essen, University of Duisburg-Essen, Hufelandstrasse 55, D-45147 Essen, Germany; Monika.Herten@uk-essen.de; 3 Department of Orthopedic, Gelenkzentrum Bergisch Land, Freiheitstraße 203, D-42853 Remscheid, Germany; Tassemeier@gelenkzentrum-bergischland.de; 4 Department of Otorhinolaryngology, University Hospital Essen, University of Duisburg-Essen, Hufelandstrasse 55, D-45147 Essen, Germany; AlexanderMaximilian.Hackel@uksh.de; 5 Department of Rheumatology and Clinical Immunology, University of Lübeck, Ratzeburger Allee 160, D-23538 Lübeck, Germany; 6 Department of Orthopedic, St. Vinzenz Krankenhaus Düsseldorf, Schloßstraße 85, D-40477 Düsseldorf, Germany; 7 Department of Trauma, Reconstruction and Orthopedic Surgery, St. Marien Hospital, Mülheim an der Ruhr, Kaiserstraße 50, D-45468 Mülheim an der Ruhr, Germany; Marcus.Jaeger@uni-due.de; 8 Orthopedics and Trauma Surgery, University of Duisburg Essen, D-45147 Essen, Germany

**Keywords:** bone marrow aspirate, mesenchymal stem cells, bone marrow aspirate concentrate, bone healing, Aspire Marrow Cellution^®^, Jamshidi needle

## Abstract

While autologous bone is still the gold standard for treatment of bone defects, its availability is limited. Sufficient numbers of mesenchymal stroma cells (MSC) may be an alternative. Small volumes of bone marrow aspirate (BMA) were harvested with two different needle systems comparing the yield and regenerative potency of the MSCs. BMA (10 mL) was aspirated from the posterior iliac crest of 12 patients with degenerative spinal disc disease using both needle systems in each patient: the Jamshidi needle (JAM) and on the contralateral side the Marrow Cellution^®^ Needle (AMC). Number of mononuclear cells (MNCs) and regeneration capacity (colony-forming unit/CFU) were determined. MSCs were characterized for surface markers and their differentiation into trilineages. There was no significant difference between the two harvesting needles regarding the quantity of MNCs in BMA: 5.2 ± 1.8 × 10^9^ MNC/mL for AMC vs. 4.8 ± 2.5 × 10^9^ MNC/mL for JAM, *p* = 0.182. The quantity of CFUs per ml BMA was similar for both groups: 3717 ± 5556 for AMC and 4305 ± 5507 for JAM (*p* = 0.695). The potency of MSCs expressed as colony-forming potential per 10^6^ MNC resulted in 0.98 ± 1.51 for AMC and 1.00 ± 0.96 for JAM (*p* = 0.666). Regardless of the needle design, 10 mL bone marrow aspirate contains a sufficient number of about 40,000 MSCs that can be used to enhance bone healing.

## 1. Introduction

In orthopedics, autologous bone grafting is the gold standard for the treatment of bone defects caused by trauma, tumor, disease or non-healing fractures. 

Autologous bone has osteoconductive properties which form a framework of bone substance. It is osteoinductive due to the cytokines and growth factors (i.e., fibroblast growth factor and bone morphogenetic protein-2) it contains and, furthermore, the mesenchymal stromal cells (MSCs) in the bone marrow make it osteogenic [1]. However, the availability of autologous bone is limited and has the disadvantage of extraction morbidity at the donor site [2,3]. In order to compensate for the lack of donor autologous bone, various bone substitute materials (BSM) such as hydroxyapatite (HA), tricalcium phosphate (TCP), calcium sulfate, calcium carbonate (CC) and bioglass have been investigated, as well as biological adhesive, which can be used in combination with cells as stem-cell-seeded scaffolds or gene-functionalized bone substitutes [4,5]. Most clinically used BSMs are based on allografts or collagen/tricalcium phosphate scaffolds [6].

Nonetheless, nearly all bone substitutes are avital or acellular. They provide a scaffold and are therefore usually osteoconductive with some osteoinductive properties, but nevertheless, delayed healing of large bone defects is still a clinical challenge. In approximately 10 percent of fractures, further surgical intervention is necessary due to impaired healing [7,8,9,10].

In order to “vitalize” BSM, the combination of synthetic bone substitute material as a scaffold and autologous bone marrow aspirate obtained from the patient’s iliac crest was already investigated in 1999, with convincing clinical healing rates in lumbar spinal fusion in 100 out of 106 patients [11].

Furthermore, the combination of synthetic bone substitutes with autologous bone marrow aspirate obtained from the patient’s iliac crest [12,13] to treat non-healing bone defects showed good results, and other clinical studies recently confirmed that the combination of scaffolds and MSCs enhances osteogenesis in long bone defects [14,15,16,17]. 

Moreover, allografts and demineralized bone matrix together with concentrated autologous bone marrow aspirate achieved good fusion rates of 84 percent (in 26 out of 31 patients) in the field of spinal surgery, especially in older patients [18].

It seems that elderly people in particular benefit from bone-enhancing therapy, as these patients suffer more frequently from fractures and the number of MSCs in the bone marrow decreases with age [19,20]. To optimize the quality of bone marrow aspirate, a technique to concentrate BMA was developed and named bone marrow aspiration concentrate (BMAC) [21].

In bone marrow aspiration concentrate (BMAC), the quantity of progenitor cells seems to be especially significant: patients with a higher number of progenitor cells had a better outcome than those with a lower number [12]. Therefore, different optimization strategies can be found in the literature to increase the number of colony-forming cells in the aspirate [22,23]. After the collection of bone marrow, there are various methods for its further processing and preparation [24,25].

However, concerns regarding the safety of BMAC products have been raised in the 15th amendment of the German Drug Law, with the result that centrifugation and concentration and thus further preparation of the aspirate for therapeutic purposes are no longer permitted in the operation room. Now, every application needs a manufacturing authorization in accordance with the German Drug Law and an accredited manufacturing laboratory. This makes the concentration procedure of BMA very expensive and clinical use unattractive as health insurance does not reimburse the cost.

**Figure A1:**
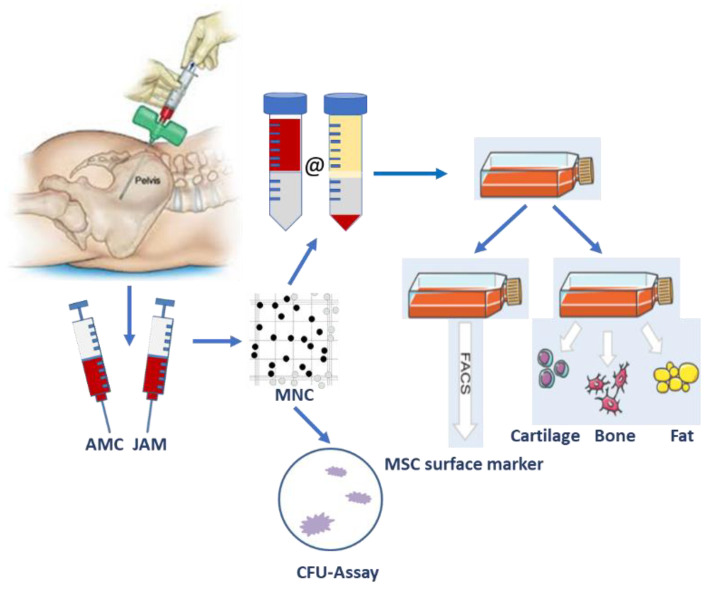
The worldwide well-known Jamshidi needle, 2. the new MarrowCellution® needle. Both systems were compared regarding the yield of mononucleated cells (MNC) and regenerative capacity via colony-forming unit (CFU) assay. Remaining BM was layered on a Ficoll gradient, the MNC separated via gradient centrifugation and cultured. Adherent cells of the 3rd passage were compared in expression for mesenchymal stroma cell (MSC) surface markers via flow cytometry and differentiation potential into the three lineages. Both systems revealed comparable amounts of MSC in 10 mL BMA. AMC, MarrowCellution® Needle; JAM, Jamshidi needle; MNC, mononuclear cells; CFU, colony-forming unit; @, gradient centrifugation; FACS, flow cytometry analysis; MSC, mesenchymal stroma cell.

Despite everything, unprocessed BMA is still allowed for nomotopic use [26]. We therefore decided to investigate whether a skillful single draw of a small volume of BMA would provide sufficient MSCs for treatment of a defect. In the present study, we investigated two methods of aspiration and compared their osteogenic potency and yield. Our results show that both needle systems provided sufficient quantities of MSCs for treatment of bone defects. It is, nevertheless, important to consider various physiological and physical aspects when harvesting BMA (Figure A1 in the Appendix A).

## 2. Materials and Methods

### 2.1. Patients

Bone marrow aspirate from 12 patients (6 male, 6 female) with the indication for mono- or multi-segmental spondylodesis using the transforaminal lumbar interbody fusion (TLIF) technique was investigated. The harvested bone marrow was partially transferred to the site of the defect—so-called homologous application without changing the essential biological properties of the graft. 

The study was approved by the local ethics committee (reference number 15-6646-BO). All subjects gave their written informed consent before surgery. The exclusion criteria were (i) age under 18 years, (ii) active or chronic tumor disease (neoplasia), (iii) acute or chronic systemic infection, (iv) pregnancy or breastfeeding.

### 2.2. Bone Marrow Aspirate Sampling

BMA was harvested under sterile conditions after surgical exposure of the spinal area before the spondylodesis procedure commenced. Harvesting took about three to four minutes. The surgical site and the order of the needle used was randomly assigned to either the Jamshidi needle (11 G, Co. Becton Dickinson BD, Heidelberg, Germany) (JAM) or the Marrow Cellution™ needle (AMC) (11 G, Aspirate Marrow Cellution, Co. Aspire Medical Innovations, München, Germany) at the contralateral iliac crest. Both needles were used according to the manufacturer’s instructions. Before use, all needle components were moistened with 2000 IU heparin/mL in 0.9% (*w*/*v*) NaCl solution (B. Braun, Frankfurt, Germany).

For both needles, the cortical bone in the area of the posterior superior iliac spine was carefully perforated. A sharp trocar was inserted, and the safe intraosseous position was checked by drawing one milliliter bone marrow aspirate with a needle under vacuum in a syringe prefilled with one milliliter heparin solution (5000 IU/mL, Ratiopharm, Ulm, Germany).

For the JAM needle, the tip was inserted under pressure and rotating movement 3–4 cm deep below the iliac crest level. The sharp trocar was then removed, the syringe was reconnected and another 9 mL of aspirate was harvested under vacuum, resulting in a total of 10 mL volume (9 mL aspirate and one ml heparin solution). The needle was not moved (no rotation and no lifting up) during the harvesting process. After harvesting was complete, the needle was pulled out.

For the AMC needle, the deep position (4 cm below level) was obtained by exchanging the sharp trocar for a blunt trocar, followed by manual pressure and rotating movements. After removal of the blunt trocar, the aspiration needle with its seven lateral openings was connected to the guide sleeve by a rotating mechanism (multiple-site method). The needle was inserted into the iliac crest and screwed clockwise until its handle reached the top of the iliac crest. The syringe was reconnected, and one milliliter of bone marrow aspirate was taken. The grip was rotated 360° counterclockwise eight times while after each rotation one milliliter of aspirate was harvested under vacuum. This counterclockwise rotation corresponds to a lifting of the aspiration needle by approximately 0.5 cm, thus ensuring a standardized aspiration technique. In total, the syringe contained a volume of 10 mL (9 mL aspirate and one ml heparin solution); then, the needle was removed.

### 2.3. Therapeutic Application

Three milliliters from each syringe (JAM and AMC aspirate) were combined with an unspecified BSM which was inserted into the lumbar disc space together with the cage of the TLIF procedure. The clinical results of the spondylodesis are not part of this study.

### 2.4. Cell Isolation and Colony-Forming Unit (CFU) Assay

Aliquots of the bone marrow aspirate were processed in the laboratory immediately after harvesting. The volume was documented, and the number of mononuclear cells counted. For this, an aliquot of the cell suspension was diluted with trypan blue and counted in a Neubauer counting chamber under a light microscope. The number of individual vital luminescent cells was determined in the squares and calculated according to the formula total cell count = (counted luminescent cells/4) × dilution factor × 10,000 × initial volume of aspirate in ml.

For the CFU assay, a serial dilution of the unprocessed aspirate with cell culture medium was performed and seeded in an MNC density of 1 × 10^5^/cm^2^, 4 × 10^5^/cm^2^ and 10 × 10^5^/cm^2^ in 6-well cell culture plates (Greiner, Frickenhausen, Germany). The cell culture medium consisted of Dulbecco’s Eagle Medium (DMEM, low glucose, Gibco, Life Technologies, Darmstadt, Germany), 1% sodium pyruvate (Sigma Aldrich, Darmstadt, Germany), 1% penicillin/streptomycin (Sigma Aldrich) and 10% fetal calf serum (FCS, Sigma Aldrich). 

The cells were cultivated at 37 °C and 5% CO_2_. After three to four days, the first medium change was performed, during which the non-adherent cells were removed. Following this, half of the medium was replaced by new medium every three to four days.

For CFU, the cells were cultivated for two weeks. Before staining, the cells were washed with PBS, fixed with 4% (*v*/*v*) formaldehyde (Merck Millipore, Darmstadt, Germany) and stained with 0.5% (*w*/*v*) Crystal Violet (Serva Electrophoresis, Heidelberg, Germany). 

The cell colonies were counted macroscopically and microscopically controlled. A colony was defined as a circular arrangement of cells derived from one colony-forming parental cell consisting of at least 50 cells. 

Cell populations are typically considered MSCs if capable of colony formation (self-replication), trilineage differentiation (along osteo-, chondro- and adipogenic mesenchymal tissue lineages) and with the expression of typical MSC cell surface markers [27]. From the CFU assay results, the number of colonies in 1 × 10^6^ MNC was determined and regarded as equivalent to the number of MSC in 1 × 10^6^ MNC. However, it should be remembered that a colony that forms from a colony-forming unit is almost always heterogeneous and contains differentiated and senescent cells [27].

The remaining bone marrow aspirate was further processed using a Ficoll gradient. For this, the aspirate was diluted with PBS (Gibco, Schwerte, Germany) containing 2% FCS (Sigma Aldrich) and layered on top of a Ficoll gradient (Ficoll Paque™ Plus, density 1.078 g/mL, GE Healthcare, Freiburg, Germany) in SepMate™ tubes (Stemcell Technologies Inc., Vancouver, Canada). After centrifugation at 1200× *g* for 10 min (acceleration 9/9, brake 7/9), the interphase (buffy coat) was recovered, washed with PBS and centrifuged for 10 min at 300× *g*. The resulting cell pellet was resuspended in cell culture medium and transferred to a T25 culture flask (Greiner) (passage 0). The cells were cultivated as described above. Before reaching 80–90% confluency, the cells were detached with Accutase (600 U/mL, Gibco/Life Technologies, Carlsbad, CA, USA), counted, diluted 1:3 and seeded in new flasks.

From the cell count of passage one and passage two, the generation time was calculated.

### 2.5. Flow Cytometric Characterization of MSCs

For flow cytometric analysis, the cells were detached, and aliquots of 0.1–0.6 × 10^6^ cells were stained with antibodies or isotype control and incubated for 30 min at 4 °C. The antibodies used were positive selection markers for the surface antigens CD105 (1:20 dilution, PE-Cy7, clone: 43A3, BioLegend), CD90 (1:200 dilution, Brilliant Violet 421, clone: 5E10, BioLegend), CD73 (1:50 dilution, PerCP-eFlour-710, clone: AD2, eBioScience) and CD29 (1:10 dilution, PE, clone: 9EG7, BD Bioscience, Heidelberg, Germany) and the negative selection markers for CD45 (1:200 dilution, V500, clone: HI30, Becton Dickinson BD Bioscience, Heidelberg, Germany) and CD34 (1:100 dilution, FITC, clone: 581, BioLegend, Fell, Germany) [28,29,30,31]. Isotype controls were diluted corresponding to the respective antibodies. After incubation, the cells were washed with 200 µl PBS, centrifuged at 460× *g* for 5 min and stained with 50 µl Fixable Viability Dye (eBioScience, eFluor® 660, 1:50 in PBS) for 30 min at 4 °C. Afterwards, the cells were measured with a flow cytometer (BD Canto II, Becton Dickinson/Bioscience, Heidelberg) and Diva Software 6.0.

### 2.6. Cell Differentiation into the Three Lineages (Osteogenic, Adipogenic, Chondrogenic)

For the analysis of the differentiation potential, the cells of the third passage were seeded in specific cell numbers according to standard protocol and incubated with differentiation medium. The medium was changed every three to four days and after 21 days the cells were stained and examined under a microscope. For all differentiation setups, respective negative controls in adjusted cell densities were seeded and cultivated in DMEM medium without any additional stimuli. All differentiation experiments and the respective controls were set up as duplicates in all 12 patients. 

In all samples, two wells each were seeded with cells with culture medium as control [32].

For osteogenic differentiation, the cells were seeded in a cell density of 5 × 10^3^/cm^2^ in 6-well cell culture plates (Fisher Scientific, Schwerte, Germany) in DMEM cell culture medium as described above. After two days, the culture medium was replaced with osteogenic differentiation medium (StemPro® Osteocyte Differentiation Basal Medium/ Osteogenesis Supplement, Fisher Scientific) with 5 µg/mL gentamycin (Sigma Aldrich)). After 21 days, the calcified extracellular matrix of the osteoblasts was stained with Alizarin red (2% (*w*/*v*) in distilled water, LifeLine Cell Technology, Oceanside, CA, USA).

For adipogenic differentiation, the cells were seeded in a density of 1 × 10^4^ cells/cm^2^ in 6-well cell culture plates. After two days of incubation, the culture medium was replaced with adipogenic differentiation medium (StemPro® Adipogenesis Differentiation Basal Medium/Adipogenesis Supplement, Fisher Scientific) in two wells. After 21 days, the fat vacuoles in the adipocytes were stained with Oil-Red-O (0.3% (*w*/*v*) in isopropyl alcohol, Sigma).

For chondrogenic differentiation, a micromass culture with 800,000 cells was seeded in 96-round bottom wells (Greiner) corresponding to a cell density of 22,860 cells/cm^2^ in chondrogenic differentiation medium (StemPro® Osteocyte/Chondrocyte Differentiation Basal Medium/Chondrogenesis Supplement, Fisher Scientific). After 21 days, the cells were stained with Alcian blue (1% (*w*/*v*) in 0.1 M hydrogen chloride, Roth, Karlsruhe, Germany) to detect the sulfated glycosaminoglycans (i.e., hyaluronic acid and chondroitin sulfate) of the chondrocytes.

### 2.7. Statistics

The statistical analysis was performed using SPSS 27.0 software (IBM Corp, Armonk, NY). Continuous variables such as cell number, number of colonies, generation time, yield per milliliter aspirate and patients’ age were summarized and presented as mean with standard deviation and/or as median with minimum and maximum. As a categorical variable, the gender distribution was shown as a percentage. Parameters such as CFU/mL aspirate were expressed as median, upper and lower median. The analysis of the normal distribution for each variable was performed using the Kolmogorov–Smirnov Test and the Shapiro–Wilk Test. For all paired, non-parametric parameters (number of MNCs, number of CFUs, MSC yield) the Wilcoxon test was used. For paired, parametric values, the paired t-test (generation time) was applied. A *p*-value < 0.05 was regarded as statistically significant.

## 3. Results

### 3.1. Patient Demographics

The study included 12 patients (6 female and 6 male). The mean patient age at the time of surgery was 62.8 ± 11.7 years (range: 43–81). 

Several comorbidities were present as was to be expected in the mostly elderly patients, with cardiovascular disease in 7 (54%), metabolic diseases in 7 (54%), pulmonary diseases in 4 (31%), depression in 2 (15%) and anemia, reflux or epilepsy in 1 (8%) case.

### 3.2. Mononuclear Cells–Yield and Generation Time

Both needle systems yielded comparable quantities of MNCs per ml with 5.3 ± 1.8 × 10^9^ for AMC and 4.8 ± 2.5 × 10^9^ for JAM (*n* = 12; *p* = 0.620) (Table 1).

After gradient centrifugation, the number of MNCs decreased by a factor of 10^2^ to 2.2 ± 1.1 × 10^7^ for AMC and 3.0 ± 2.0 × 10^7^ for JAM (*n* = 8). The average time to cell doubling was shorter in passage 1 in the AMC group with 15.1 ± 8.9 vs. 16.8 ± 12.2 days for JAM with a strong trend toward significance (*p* = 0.057). For passage 2, the generation time was comparable, with 11.7 ± 6.5 days vs. 11.7 ± 4.6 days for AMC and JAM (*p* = 0.942).

The quantity of CFUs per ml BMA was similar for both groups, 3717 ± 5556 (median 1809) for AMC and mean 4305 ± 5507 for JAM (median 2608) for both groups (*p* = 0.695).

The cells of both needle systems fulfilled the criteria for harvest of mesenchymal stroma cells according to the International Society for Cellular Therapy (ISCT). The CFU assay per 10^6^ MNC resulted in 0.98 ± 1.51 for AMC and 1.00 ± 0.96 for JAM (*p* = 0.666). There was no significant difference between the two needle systems with regard to the quantity and potency of MSCs.

### 3.3. Regeneration Potential Based on the CFUs 

**Figure 1:**
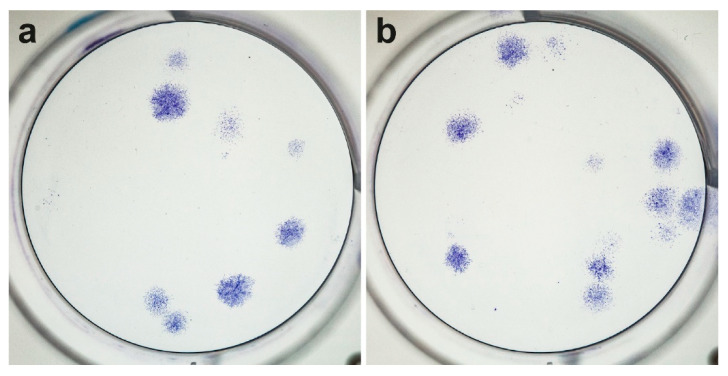
Colony-forming units (CFU). Exemplary representation of colony-forming cells seeded in a density of 1 × 10^5^/cm^2^, 4 × 10^5^/cm^2^ and 10 × 10^5^/cm^2^ in 6-well cell culture plates. After incubation for 14 days, the cells were stained with crystal violet and a colony was defined as a circular arrangement of cells consisting of at least 50 cells. (**a**). AMC, (**b**). JAM, both in a density of 10 × 10^5^ cells/cm^2^).

**Figure 2:**
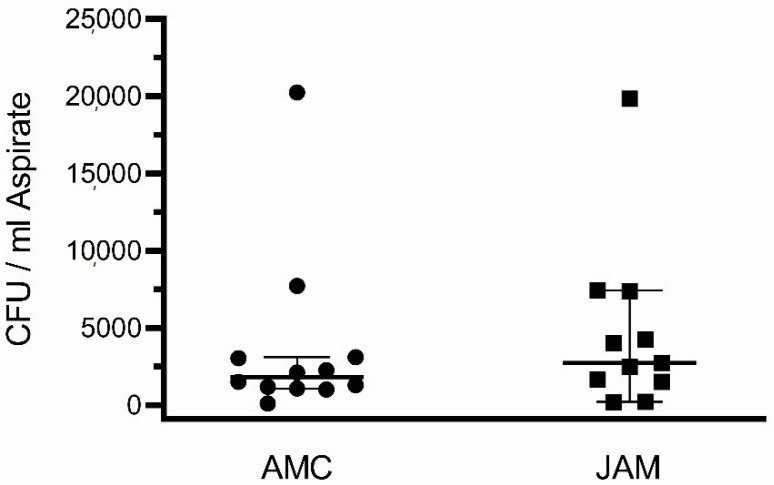
Number of colony-forming units (CFU) per ml of bone marrow aspirate. The single values are displayed as dots (AMC, median 1809) (*n* = 12) or squares (JAM, median 2608) (*n* = 11) for each patient. The median is presented as a horizontal line with a confidence interval (CI) 5–95%.

After 14 days, CFUs were detected in 12 patient samples and in both groups (AMC and JAM) (Figure 1). The average number of colony-forming units (CFU) per ml BMA did not differ between the groups with 3717 ± 556 for AMC with a median of 1809 and 4305 ± 5507 for JAM with a median of 2608 (*p* = 0.695) (Figure 2). 

### 3.4. Flow Cytometric Analysis and Differentiation Potential

**Figure 3:**
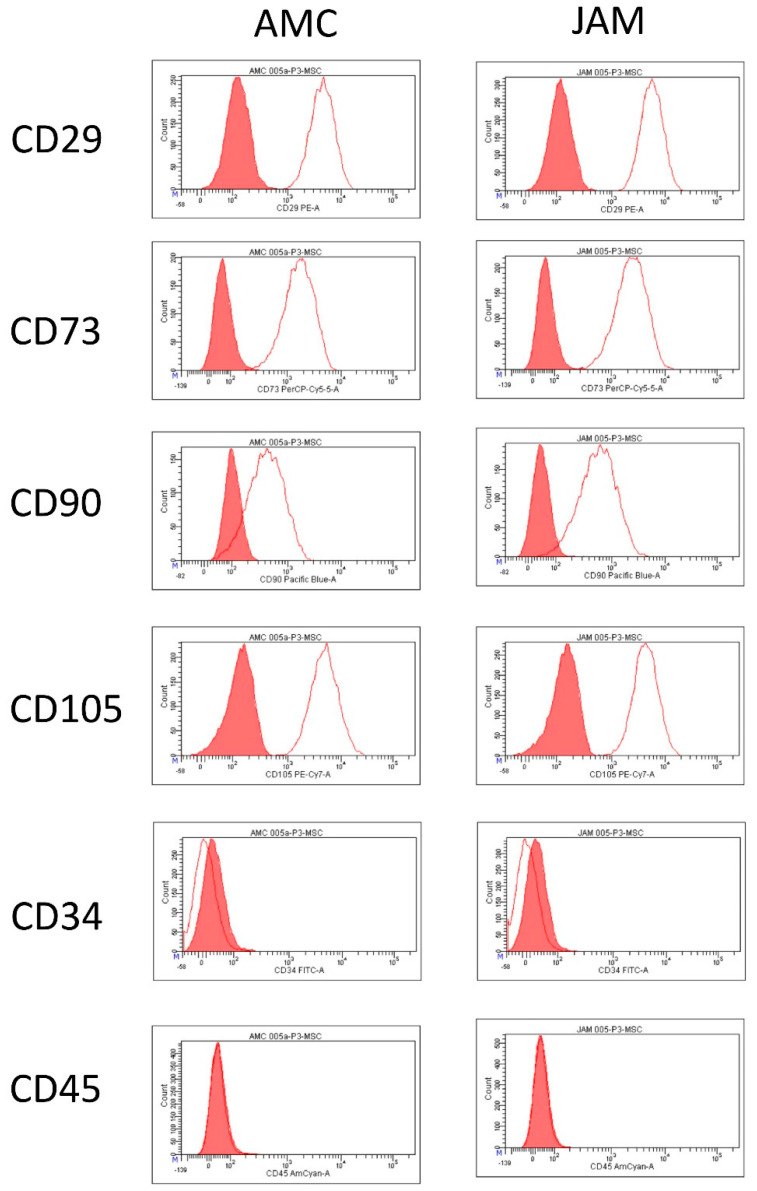
Exemplary representation of a flow cytometric analysis of a sample. The horizontal axis indicates the intensity of the individual measurement and the vertical axis indicates the number of cells. All single histograms show the isotype control in red and the positive dataset as a red line, allowing the positive cells to be accurately identified.

**Figure 4:**
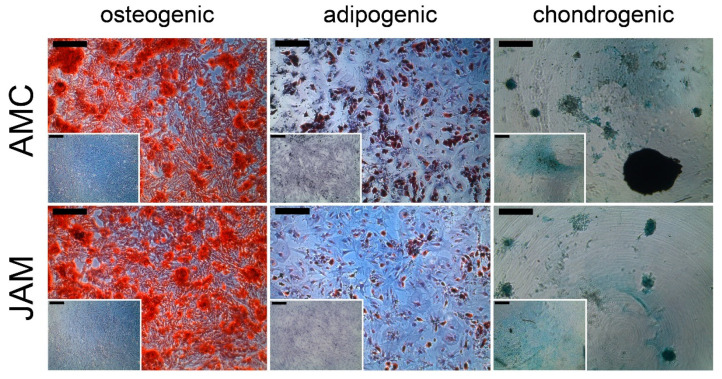
Differentiation into the three lineages. Exemplary presentation of osteogenic, adipogenic and chondrogenic (left to right) differentiation, each with the respective control without differentiation medium. The small black background bar represents 200 µm.

All cells of the third passage were analyzed and found to be positive for the markers CD29, CD73, CD90, CD105 and negative for the hematopoietic markers CD34 and CD45. There were no differences between the two systems AMC and JAM in the expression of the surface markers (Figure 3). Additionally, all cells could be differentiated into the osteogenic, adipogenic and chondrogenic lineage (Figure 4).

## 4. Discussion

This study shows that a small volume of bone marrow harvested with needles of two different designs without further processing is sufficient to obtain enough MSCs for clinical use in bone defect surgery.

Data revealed comparable quantities of CFUs per milliliter BMA for both needles with an average of 3717 for AMC and 4305 for JAM. Regarding the number of CFUs derived from one million MNCs, similar results for the CFU assay per 10^6^ MNC were found, with 0.98 for AMC and 1.00 for JAM.

In all patient samples, whether taken from the AMC or JAM group, the harvested cells adhered to plastic surfaces, stained positive for the surface markers CD29, CD73, CD90, CD105 and negative for the hematopoietic markers CD34 and CD45 and were able to differentiate into the three lineages in respective differentiation medium. Therefore, they fulfilled the criteria of mesenchymal stroma cells without showing a significant difference between the two groups.

Although the present results do not show that there is a statistically significant superior yield of mesenchymal stromal cells using the AMC needle compared to the JAM needle, there is a tendency in favor of the AMC technique. However, this tendency was not statistically relevant and may be due to the relatively small sample size.

### 4.1. Previous Experience with the New AMC Needle

In 2019, a study compared the bone marrow aspirate harvested with the AMC needle (multiple-site method) with concentrated bone marrow aspirate (BMAC) from the contralateral side of the iliac crest in 30 patients with an average age of 56 years (range: 21–85 years) [33]. In respect to the number of MNCs per ml, our results showed a slightly lower number of MNCs (22.5 × 10^6^ AMC/29.7 × 10^6^ JAM) than Scarpone et al. with 35.2 × 10^6^ using the AMC needle. Concerning the colony-forming units (CFU), the authors found an average number of CFU 2885 ± 1716 per ml with the AMC needle [33]. In the present study, slightly higher CFU counts per ml aspirate with an average of 3,717 for AMC and 4305 for JAM were detected, although with a high range of variation (98 to 23,725).

### 4.2. Multiple-Site Drawing Method

Regarding the drawing method, Oliver et al. compared a single-site to a multiple-site method using the standard Jamshidi needle in 2017. In six patients, 51 mL of bone marrow was drawn, either in one draw (single-site) or by pricking six times (multiple-site method) on the contralateral site. Bone marrow aspirate was concentrated to a final volume of 6.5 mL. The number of MNCs in the BMAC was slightly higher using the multiple-site method (31 vs. 23 × 10^6^ mL), whereas the resulting MSC quantity was higher using the single-site method (3486 vs. 2722/mL concentrate) [34]. Compared to our data, even without using a concentration system, we harvested comparable quantities of MNCs/mL aspirate compared to their concentrate and slightly higher values for MSCs per ml aspirate.

### 4.3. Comparing Harvesting Techniques

Concerning the harvesting technique, Jäger et al. proposed in 2010 that inserting the needle into the iliac bone using a divergent technique achieves better results than a parallel technique [35]. 

Furthermore, it is known that there is a negative correlation between the volume drawn, the size of the syringe used for drawing and the yield of MSCs [15,36].

In a recent study, 52 mL bone marrow was aspirated with a 30 mL syringe in 90 patients (mean age 54 years (range: 18–80)) using the divergent technique and concentrated to 7 mL BMAC. The CFU number reached 37–57 CFUs/mL BMAC, which was much less than in our approach. [37]. 

In a study with 41 patients (mean age 44 years (range: 14–66)), a high volume (125 mL per iliac side, in total 252 mL) of bone marrow was harvested with a 20 mL syringe and revealed 16 × 10^6^ MNCs and 214 CFUs per ml aspirate [38]. Their yield, especially the number of CFUs, was much smaller compared to our results. As a method to increase the number of bone precursor cells in the puncture, Muschler et al. recommend small aspiration volumes. They showed that four one-milliliter aspirates will provide almost twice the number of colony-forming units as one four-milliliter aspirate [36].

In a study with a cohort of 30 patients, Hernigou et al. showed that the amount of progenitor cells was 300 percent higher in the aspirate taken by a 10 mL syringe than in the aspirate taken with a 50 mL syringe [22].

Piuzzi et al. also recommend taking small aspiration volumes (1 to 2 mL) from different positions in the bone with a respective distance of 5 to 10 mm [22,23]. 

### 4.4. Further Processing of the Aspirate

Further processing of the aspirate does not seem to be necessary to increase the number of MSCs in the bone marrow aspirate. In various studies, a higher volume of bone marrow was drawn and resulted in fewer MSCs after centrifugation. Kevy et al. aspirated 120 mL bone marrow, concentrated the volume to 20 mL and gained about 61,000 progenitor cells. In the present study, in contrast, 10 mL aspirate provided 37,000 progenitor cells using the AMC needle and 43,000 using the JAM needle without further processing. Doubling the aspired volume does not seem to convert to a doubling of cell numbers [39]. 

This was confirmed in a further study, where Shapiro et al. harvested 26 mL from each iliac crest side (52 mL in total) in 25 patients (mean age 60 years (range: 42–68)). After centrifugation, a concentration volume of 6 mL was collected with a median of 34,400 (435–1,449,000) cells in total [40]. In this regard, the concentration system used did not appear to affect the number of progenitor cells obtained. Hedge et al. showed that comparison of three different concentration systems for 60 mL bone marrow aspirate in 40 patients (mean age 47 years (range: 18–92)) resulted in comparable amounts. The highest values were achieved with the Harvest SmartPReP 2 BMAC system, resulting in 7 mL concentrate with 1270 CFUs/mL, which corresponds to less than one third of our yield [21]. 

### 4.5. Increasing the Local Concentration of MSCs

In the surgical treatment of poorly healing bone tissue, different methods have been developed for obtaining and increasing the local concentration of MSCs. This is because the regeneration potential correlates significantly with the number of osteoprogenitors in the aspirate [32]. 

As far as the optimal aspiration site is concerned, i.e., the location where the largest number of MSCs can be found, several studies state that the iliac crest provides more MNCs with a range of 25 to 55 × 10^6^/mL than other bone marrow locations (i.e., humerus, femur, tibia, vertebral body and calcaneus) with a range of 6 to 39 × 10^6^/mL [41,42,43]. 

As discussed above, there are various suggestions regarding the factors which influence the cell count in the aspirate: the choice of syringe size, the volume taken and the technique, and the further processing of the aspirate, such as concentration. 

Regarding syringe size, the use of a 10 mL syringe is widely recommended. In the present study, 10 mL syringes were used for both techniques. Directly below the cortical level of the anterior iliac crest, one milliliter was aspirated with the Jamshidi needle while applying a vacuum with a 10 mL syringe. A further 8 mL was obtained 3–4 cm below the cortical level. This was a double-site aspiration. The AMC needle was used on the contralateral iliac crest of the same patient in prone position. In contrast to the Jamshidi needle, the AMC needle, due to its design, aspirated from several areas via the lateral openings (multiple-site aspiration).

Furthermore, several studies have shown that there is a clear correlation between sample quality, surgeon experience, technique and needle [35,44,45]. Since the number of MSCs decreases when aspiration is repeated at the same location, it is recommended that the location of harvesting should be changed at least after every third puncture in order to obtain the highest possible yield of MSCs [35]. The AMC needle with its multiple-opening sites fulfils these demands. Hence, we expected that the AMC would yield more MSCs because with every rotation there is a change of location. As this was not the case, we suspect that there are other factors which play a role. 

The vacuum pressure to release the MSCs could be one factor that influences the success of bone marrow aspiration, since MSCs are attached to bone [22]. Jäger and Hernigou reported in 2010 that the geometry of the aspiration syringe has an influence on the yield of MSCs in the aspirate. The pressure required to aspirate the mesenchymal cells is applied at the tip of the needle and is defined by the formula: Pressure (P) = Force (F)/Area (A). Narrow, long syringes are therefore preferable when collecting MSCs by bone marrow aspiration [35].

Moreover, Gronkjaer et al. compared whether a gentle pull of the needle plunger or a rapid jerk of the plunger, which creates a rapid negative pressure, increases the yield of MSCs. They concluded that a jerky movement of the piston transported about twice as many cells as the gentle pull. This can be explained by the fact that the less viscous blood flows first and the more viscous marrow remains behind or flows more slowly. A quick jerk on the plunger creates a higher difference in pressure at the lumen of the needle in the medullary cavity. This rapid change in pressure prevents the faster flow of blood into the needle [46]. 

To further increase the cell density of bone marrow aspirates, centrifugation techniques promising higher yields were developed 10 to 15 years ago [13,47]. For reasons of cost, the lack of reimbursement for clinical use in Germany and finally also due to regulatory concerns of the Paul Ehrlich Institute, the techniques did not become established in daily clinical practice. This has been classified by the Paul Ehrlich Institute as a "drug of concern" and according to paragraph § 5 (1) German Drug Law it is prohibited to place questionable drugs on the market or use them on another person. Nevertheless, there seems to be no difference between centrifuged aspirate and native aspirate, at least with regard to differentiation, immune modulation and angiogenesis as shown in a study with 33 patients [25]. 

### 4.6. Increasing Clinical Relevance of Cell-Based Therapies with Bone Marrow Stromal Cells 

Cell-based therapies with bone marrow stromal cells are playing an increasingly important role in bone regeneration. On PubMed, the MeSH term “bone marrow mesenchymal stroma cells” revealed 726 publications. Starting in 1975, the development over the years was *n* = 35 until 1995, *n* = 132 in the period 1996–2005 and *n* = 562 between 2006 and 2020. Combination of the search terms “bone marrow” and “bone regeneration” revealed more than 10,000 publications between 2000 and 2020. In the first decade of this period only half as many publications appeared as in the second from 2010 to 2020. Currently, there are 21 ongoing clinical trials matching the search term “bone marrow concentrate” on clinicaltrials.gov. Most of them are investigating (i) BMAC in osteoarthritis (*n* = 11), (ii) fractures and other bone defects (*n* = 4), (iii) spinal fusion and discogenic pain (*n* = 6), (iv) other non-bone-related uses of BMAC (*n* = 27). 

This evaluation of the literature search clearly demonstrates that interest in regenerative therapies in orthopedics and trauma surgery is steadily increasing and the requirements for intelligent products are growing. It was recently demonstrated that an orthopedic suction handle concentrates significant quantities of MSCs during surgery, which may be a promising clinical application in the future [48].

### 4.7. How Many Cells Are Required to Heal a Bone Defect?

Hernigou et al. treated 60 patients with atrophic non-union of the tibia with an injection of 20 mL BMAC from the iliac crest containing on average 2579 ± 1121 progenitors/mL (60–6120 progenitors/mL). After four months, bone union was reached in 53 (88%) of the patients whose BMAC contained >1500 progenitors/mL and an average total of 54,962 ± 17,431 progenitors. Non-union was found in the patients with significantly lower numbers of injected progenitor cells [49].

Dai et al. successfully augmented spinal fusion with β-TCP in 39 out of 41 patients with autologous BMAC containing about 40,000 MSCs (45 mL concentrate from 252 mL bone marrow aspirate) [38]. 

However, individual factors such as comorbidities should also be considered. A study from 2016 showed that diabetics, compared to non-diabetics, have worse non-union healing despite treatment with the same number of autologous MSCs (76% diabetics vs. 91% non-diabetics) [50].

As demonstrated in the studies above, the critical number of MSCs that are necessary to heal a defect is evaluated and discussed differently. El-Jawhari et al. showed that there is a positive correlation between white blood cells in the aspirated bone marrow and the capacity for colony formation. 

In addition to MSCs, there are other cells and secreted factors as well as the selection of biomaterials that may play a crucial role in osteogenesis and regeneration [25,51]. 

### 4.8. Limitations of the Present Study

The study included only 12 patients, which had an impact on the statistical power of the test procedures chosen. A larger cohort might have statistically demonstrated the advantage of the AMC needle for the yield of mesenchymal stromal cells, even though both techniques were applied on each patient. However, only a small bias is to be expected since the removal technique using the AMC and Jamshidi needles was precisely applied by the two surgeons involved in the study, and the side of the iliac crest was randomly assigned to the needle type. In addition, the risk of bias in the results was reduced by keeping the aspirate volume constant on both sides.

## 5. Conclusions

The search for the ideal method of obtaining therapeutically useful MSCs continues. The challenges of bone marrow aspiration continue to be harvesting the ideal volume, placing the needle in the optimal area of the bone, finding the best vacuum setting and probably making the right decision for the perfect aspiration system. Nevertheless, we demonstrated that, regardless of the needle system used, 10 mL bone marrow aspirate contains a sufficient number of about 40,000 MSC that can be used to enhance bone healing.

## Figures and Tables

**Table 1:** Yield of mononuclear cells (MNC)/mL in bone marrow aspirate harvested with the AMC or the JAM needle system before gradient centrifugation.

Patient	1	2	3	4	5	6	7	8	9	10	11	12
AMC MNC × 10^9^	4.3	5.1	2.5	5.1	6.2	7.2	5.7	6.8	8.8	4.0	3.6	3.9
JAM MNC × 10^9^	8.5	3.3	2.0	3.3	4.6	4.0	4.9	9.9	7.6	3.1	3.1	3.6

## Data Availability

The raw data are available from the corresponding author on reasonable request.
